# A Narrative Review of Augmentation Strategies in Obsessive-Compulsive Disorder: Antipsychotics as Mainstay and Emerging Role of Extended-Release Methylphenidate

**DOI:** 10.3390/ph19040551

**Published:** 2026-03-30

**Authors:** Julija Grigaitytė, Robertas Strumila

**Affiliations:** Psychiatric Clinic, Faculty of Medicine, Institute of Clinical Medicine, Vilnius University, LT-03101 Vilnius, Lithuania; robertas.strumila@mf.vu.lt

**Keywords:** obsessive-compulsive disorder (OCD), augmentation strategies, treatment-resistant OCD, antipsychotics, extended-release methylphenidate (MPH-ER), pharmacotherapy, dopamine receptor binding affinity

## Abstract

Obsessive-compulsive disorder (OCD) is a chronic mental disorder characterized by distressing thoughts and repetitive behaviors that significantly impair daily functioning and quality of life. Many patients fail to achieve sufficient symptom relief with first-line treatments, such as cognitive-behavioral therapy (CBT) or selective serotonin reuptake inhibitors (SSRIs). Dopaminergic dysregulation has been implicated in the pathophysiology of OCD, providing a rationale for pharmacological augmentation strategies. This article presents a narrative review of the evidence regarding the efficacy, safety, and clinical applicability of antipsychotic agents and emerging pharmacological augmentation approaches, including extended-release methylphenidate (MPH-ER), in SSRI-resistant OCD. A literature search was conducted using PubMed, EBSCO, and Embase databases, with an additional search of Google Scholar, focusing on studies examining pharmacological augmentation in treatment-resistant OCD. Overall, the evidence base is limited by small sample sizes, short follow-up durations, heterogeneous response criteria, and a lack of head-to-head comparisons versus CBT augmentation, which constrains the generalizability of conclusions. Dopamine receptor antagonists, particularly risperidone, as well as the partial agonist aripiprazole, remain the most consistently supported augmentation strategies, while olanzapine and quetiapine may be considered in selected cases. Evidence for MPH-ER is currently limited—supported by one small RCT and two recent case series—and may be considered in carefully selected adults with comorbid ADHD or marked executive dysfunction, although larger controlled studies and long-term safety data are required before firm clinical recommendations can be made.

## 1. Introduction

Obsessive-compulsive disorder (OCD) is a chronic and disabling neuropsychiatric condition, characterized by recurrent, intrusive thoughts, images, or urges (obsessions) and repetitive behaviors or mental acts (compulsions) performed in response to these obsessions. These symptoms are typically time-consuming, cause significant distress, and substantially impair daily functioning, social relationships, and occupational performance. Epidemiological studies estimate the lifetime prevalence of OCD at approximately 1–3% in the general population, making it a relatively common psychiatric disorder [1]. Onset frequently occurs in childhood or adolescence, often persisting into adulthood, and the disorder typically follows a waxing-and-waning, but chronic course [2]. Early-onset OCD, occurring before age 10, is more common in males, while females are at greater lifetime risk of developing the disorder, usually during adolescence [3].

The impact of OCD extends beyond the individual, affecting family members and caregivers. Families often accommodate patients’ compulsions, participating in repetitive rituals or providing reassurance, which can become burdensome and impair the family functioning [4]. OCD is linked to substantial socioeconomic consequences, such as higher rates of unemployment, disability, and treatment-related financial burden, and is recognized by the World Health Organization (WHO) as one of the ten most disabling illnesses worldwide. Despite its significant burden, OCD is frequently underdiagnosed and undertreated, with many patients experiencing prolonged delays [1].

**Figure 1:**
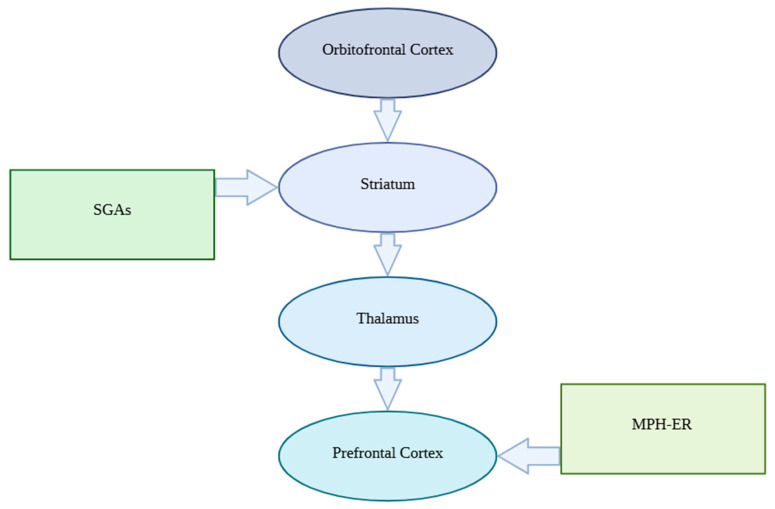
Dopaminergic modulation of CSTC circuitry in SSRI-resistant OCD.

OCD is a heterogeneous disorder with complex neurobiological underpinnings. Neuroimaging and neurochemical studies indicate dysfunction within cortico-striato-thalamo-cortical (CSTC) circuits, including the orbitofrontal cortex (OFC), anterior cingulate cortex (ACC), prefrontal cortex (PFC), caudate nucleus, and thalamus. Hyperactivity within these circuits is thought to contribute to repetitive thoughts and compulsive behaviors. Aberrant connectivity has also been observed in broader associative networks, including limbic structures, parietal cortex, and cerebellum. Dysregulation of serotonergic, dopaminergic, and glutamatergic neurotransmission is implicated in these circuit abnormalities. Chronic SSRI administration modulates dopaminergic tone in CSTC circuits, providing a rationale for dopamine-targeted augmentation strategies in treatment-resistant cases [5]. The therapeutic effect of antipsychotic augmentation is thought to arise from modulation of striatal dopamine transmission and serotonergic receptor blockade. Hyperdopaminergic signaling within CSTC circuits supports the rationale for augmentation with second-generation antipsychotics (SGAs), which act primarily as D2/D3 receptor antagonists or partial agonists to reduce maladaptive habitual responding. Conversely, presynaptic dopamine enhancers such as extended-release methylphenidate (MPH-ER) may improve prefrontal executive control over intrusive thoughts by enhancing dopaminergic tone in the PFC (Figure 1). Together, these pharmacological approaches represent complementary strategies to modulate CSTC circuit dysfunction in SSRI-resistant OCD [6,7]. Interindividual variability in efficacy and tolerability may be partly explained by pharmacogenomic factors: antipsychotics such as risperidone and aripiprazole are primarily metabolized by CYP2D6 and CYP3A4, and genetic variation in these enzymes can influence plasma concentrations, prolactin elevation, and metabolic or neurological adverse effects [8,9]. Extended-release methylphenidate is predominantly metabolized by carboxylesterase-1 (CES1), and functional CES1 variants have been shown to significantly influence methylphenidate exposure and clinical response [10]. In addition, polymorphisms in catechol-O-methyltransferase (COMT), a key regulator of dopamine degradation in the prefrontal cortex, may modulate dopaminergic signaling and executive functioning, potentially affecting treatment response to stimulant augmentation, particularly in patients with comorbid ADHD or executive dysfunction [11,12]. Together, these molecular and genetic mechanisms provide a biologically plausible explanation for heterogeneous treatment outcomes and support the future integration of pharmacogenomics-informed, personalized augmentation strategies in treatment-resistant OCD.

The diagnosis of OCD is based on clinical assessment according to the Diagnostic and Statistical Manual of Mental Disorders, Fifth Edition (DSM-5), which requires the presence of obsessions, compulsions, or both. Obsessions are recurrent, persistent thoughts, urges, or images experienced as intrusive and unwanted, typically causing marked anxiety or distress. Compulsions are repetitive behaviors or mental acts performed to neutralize the distress associated with obsessions or to prevent a feared outcome, although they are not realistically connected to the event they aim to prevent [13]. Definitions of response, remission, and refractoriness vary across studies. Expert consensus criteria categorize response stages based on Yale-Brown Obsessive-Compulsive Scale (Y-BOCS) scores. Full response is generally defined as a ≥35% reduction in Y-BOCS scores after an adequate trial of first-line therapy, partial response as 25–35%, and non-response as <25%. Treatment-resistant or refractory OCD refers to patients who fail to achieve adequate improvement following multiple trials of first-line interventions [14].

First-line treatment for OCD includes both pharmacological and psychological interventions. SSRIs, including fluoxetine, sertraline, paroxetine, fluvoxamine, citalopram, escitalopram, and clomipramine, have demonstrated efficacy in numerous randomized controlled trials (RCTs), typically lasting 8–12 weeks at maximum tolerated doses. CBT, particularly exposure and response prevention (ERP), is equally effective, and combination therapy is recommended for severe or treatment-resistant cases. Despite these first-line treatments, 40–60% of patients fail to achieve full symptom remission, and residual symptoms may continue to significantly impair functioning [15].

For patients with inadequate response to SSRIs or CBT, augmentation strategies have been investigated. Augmentation involves adding medications with complementary mechanisms to enhance therapeutic response. Antipsychotics, particularly risperidone and aripiprazole, are the most well-documented augmentation agents [16]. The neurobiological rationale for antipsychotic augmentation lies in the modulation of dopaminergic hyperactivity within CSTC circuits, normalization of reward and learning pathways, and improvement of top-down cortical inhibition of limbic structures. Neuroimaging studies indicate that antipsychotic therapy can improve dysfunction in the anterior cingulate and prefrontal cortices, enhancing the regulation of intrusive thoughts and compulsive behaviors. Interactions between serotonergic and dopaminergic systems are complex, and precise mechanisms remain under investigation. Additionally, Ducasse et al. (2014) found that antipsychotic efficacy in treatment-resistant OCD is linked to D2 and D3 receptor affinity, which may guide targeted therapy when standard SSRI treatment proves insufficient [17].

Methylphenidate (MPH), a medication commonly used for attention-deficit/hyperactivity disorder (ADHD), has recently emerged as a potential augmentation strategy in treatment-resistant OCD. Stimulants, including MPH, may enhance dopaminergic neurotransmission in the prefrontal cortex, improving executive functions such as response inhibition, attention allocation, and cognitive flexibility, which may reduce distraction from intrusive thoughts and facilitate engagement with non-OCD tasks [18,19]. Evidence for MPH-ER remains limited, with reports of symptom worsening in some patients, underscoring the need for careful, individualized use and further large-scale studies to establish efficacy and long-term safety. Overall, targeted dopaminergic modulation may complement serotonergic therapy and improve outcomes in select patients with refractory OCD [20].

Note on search strategy: literature search was primarily keyword-based in PubMed, EBSCO, and Embase, supplemented with Google Scholar. MeSH terms or controlled vocabulary were not systematically used; this may have limited capture of some relevant studies, representing a potential methodological limitation.

Given this background, this article analyzes studies on augmentation approaches in OCD and provides evidence-based recommendations for their clinical application.

## 2. Results

### 2.1. First-Generation Antipsychotics

First-generation antipsychotics (FGAs), particularly haloperidol, have been evaluated as augmentation strategies in obsessive-compulsive disorder (OCD), primarily in patients with inadequate response to selective serotonin reuptake inhibitors (SSRIs). Early randomized trials suggested that low-dose haloperidol augmentation (typically 2–4 mg/day) could produce significant reductions in Y-BOCS scores in a subset of treatment-resistant patients, particularly those with comorbid tic disorders [21]. However, these studies were generally small and conducted before the widespread use of second-generation antipsychotics. The clinical use of FGAs is limited by poor tolerability and a high risk of extrapyramidal side effects. For this reason, FGAs are considered only when second-generation antipsychotics (SGAs) are contraindicated, and should be prescribed cautiously at low doses with slow titration [22]. Given these limitations, greater emphasis in research is placed on SGAs, which demonstrate a more favorable balance of efficacy and tolerability in treatment-resistant OCD.

### 2.2. Second-Generation Antipsychotics

Augmentation with SGAs represents the most established pharmacological approach for treatment-resistant OCD. Compared with FGAs, SGAs offer a broader receptor profile and a more favorable risk–benefit balance. Evidence indicates that risperidone and aripiprazole are among the most effective options, while the efficacy of other atypical agents such as olanzapine and quetiapine remains less consistent [23]. In this context, the following section will specifically review these four main agents: risperidone, aripiprazole, olanzapine, and quetiapine.

#### 2.2.1. Aripiprazole

Aripiprazole has consistently been reported as an effective augmentation strategy in treatment-resistant OCD. Across clinical studies, aripiprazole is typically initiated at low doses (5 mg/day) and titrated gradually to 10–15 mg/day depending on clinical response and tolerability. Its pharmacological profile, acting as a dopamine D2 receptor partial agonist and a 5-HT1A receptor partial agonist, classifies it as a dopamine-serotonin system stabilizer, which may underlie its therapeutic benefits in OCD patients who fail to respond to standard SSRIs or clomipramine [24]. Compared with other SGAs, aripiprazole is often promising due to its relatively favorable side-effect profile [25].

Open-label studies support these findings. Delle Chiaie et al. (2011) treated 20 SSRI- or clomipramine-resistant patients with aripiprazole doses titrated from 5 mg/day up to 20 mg/day. After 12 weeks, patients demonstrated a significant decrease in Yale-Brown Obsessive-Compulsive Scale (Y-BOCS) scores (*p* = 0.0001), with side effects generally mild and including akathisia, tremor, nausea, and fatigue [26]. In a similar 10-week study, Ak et al. (2011) observed reductions in both total and subscale Y-BOCS scores in 30 SRI-resistant patients, with 30% meeting the responder criterion [27].

Case reports further illustrate its potential in complex clinical presentations. Izci et al. (2016) reported a series of five patients with treatment-resistant OCD who achieved substantial symptom reduction within 4–6 weeks of aripiprazole augmentation (10–30 mg/day) added to clomipramine, accompanied by improvements in both obsessive-compulsive and anxiety symptoms [28].

Randomized controlled trials provide more rigorous evidence. Sayyah et al. (2012) demonstrated that 12 weeks of aripiprazole (10 mg/day) augmentation significantly reduced Y-BOCS scores in 39 adults with SSRI-resistant OCD compared with placebo, while Muscatello et al. (2011) showed that 15 mg/day aripiprazole over 16 weeks improved not only Y-BOCS scores but also certain cognitive functions, including attentional control and executive function [29,30]. Comparative trials further support its clinical value: Assarian et al. (2016) found aripiprazole more effective than risperidone in reducing Y-BOCS scores among 100 treatment-resistant patients [31]. In a 16-week open-label study, Dar et al. (2021) reported that aripiprazole (15 mg/day) and olanzapine (10 mg/day) improved Y-BOCS scores in 60 treatment-resistant patients, while L-methylfolate did not show significant benefits [32].

Overall, across case reports, open-label trials, and controlled studies, aripiprazole appears to be a valid and generally well-tolerated adjunct in treatment-resistant OCD, with typical effective doses ranging from 10 to 15 mg/day. Nevertheless, given the relatively small sample sizes and short follow-up periods of most studies, further research is warranted to establish long-term efficacy, optimal dosing, and safety.

#### 2.2.2. Risperidone

Risperidone is among the best-supported antipsychotic augmentation strategies for SSRI-resistant OCD [33]. In most trials, risperidone was used at relatively low doses (0.5–3 mg/day), substantially lower than doses commonly used for psychotic disorders, reflecting its role as an adjunctive rather than primary antipsychotic treatment.

Early double-blind, placebo-controlled trials demonstrated its efficacy and tolerability. McDougle et al. (2000) randomized 36 adults with SSRI-resistant OCD to receive adjunctive risperidone (mean dose 2.2 mg/day) or placebo for six weeks. In the risperidone group, 50% of patients met responder criteria, compared with none in the placebo group, and treatment was generally well tolerated, with rapid and sustained reductions in obsessive-compulsive symptoms [34]. Hollander et al. (2003) conducted a double-blind, placebo-controlled trial involving 16 patients with SSRI-refractory OCD, randomized to receive risperidone (0.5–3.0 mg/day; *n* = 10) or placebo (*n* = 6) over eight weeks. Forty percent of patients in the risperidone group met response criteria (≥25% reduction in Y-BOCS), compared with none in the placebo group. Risperidone was generally well tolerated, with only one discontinuation due to adverse effects, further supporting its efficacy as an augmentation strategy in this population [35].

Longer-term studies have also explored its effectiveness. Matsunaga et al. (2009) evaluated 44 SSRI-refractory patients randomized to receive risperidone, olanzapine, or quetiapine as add-on therapies, combined with cognitive-behavioral therapy (CBT) over a 12-month period. Significant improvements in Y-BOCS scores were observed, although outcomes remained less favorable than in patients responsive to SSRIs plus CBT alone, and long-term use raised some safety considerations [25].

Maina et al. (2008) compared risperidone (1–3 mg/day) with olanzapine (2.5–10 mg/day) over eight weeks in a single-blind trial of 50 treatment-resistant patients, finding similar reductions in Y-BOCS scores; however, risperidone was associated with amenorrhea, while olanzapine led to weight gain, highlighting the need for careful monitoring of adverse effects [36]. As mentioned earlier, Assarian et al. (2016) directly compared risperidone (1.5 mg/day) with aripiprazole (5 mg/day) in 100 treatment-resistant patients. Both drugs significantly reduced Y-BOCS scores, although risperidone produced somewhat smaller improvements than aripiprazole, suggesting that while risperidone is an effective augmentation option, aripiprazole may offer greater symptom reduction in similar clinical populations [31].

In general, risperidone remains a key evidence-based option, particularly when carefully titrated and monitored.

#### 2.2.3. Olanzapine

Olanzapine’s high affinity for both 5-HT2 and D2 receptors has made it a candidate for augmentation in OCD [37]. Clinical studies have generally used doses ranging from 2.5 mg/day to 10 mg/day, with gradual titration depending on symptom response and metabolic tolerability [38].

Evidence regarding its efficacy in treatment-resistant OCD is limited and mixed. Bystritsky et al. (2004) conducted a double-blind, placebo-controlled trial with 26 patients receiving adjunctive olanzapine (up to 20 mg/day) or placebo for six weeks. The olanzapine group showed a significant reduction in Y-BOCS scores, with 46% achieving ≥ 25% improvement, whereas none in the placebo group met this threshold [39]. Similarly, as previously discussed, Maina et al. (2008) found that olanzapine was effective, showing comparable reductions in Y-BOCS scores to risperidone, while also highlighting its tendency to cause weight gain [36]. Weiss et al. (1999) in 10 SSRI-resistant OCD, and Bogetto et al. (2000) in 23 fluvoxamine-refractory patients both reported improvements in Y-BOCS scores with olanzapine augmentation [40,41]. Therefore, olanzapine may be considered in selected cases, but its use requires careful metabolic monitoring and individualized risk–benefit assessment.

Long-term data from Matsunaga et al. (2009) showed that adjunctive olanzapine over 12 months produced significant Y-BOCS reductions alongside CBT, but improvements were smaller compared to patients responsive to SSRIs plus CBT, and safety concerns such as weight gain and metabolic effects were noted [25]. Similarly, the Italian long-term open-label study by Marazziti et al. (2005) investigated olanzapine augmentation in 26 treatment-resistant OCD outpatients already on SRIs. After 12 weeks, most patients showed reductions in Y-BOCS scores, which were maintained over 12 months, with only mild side effects reported, suggesting that olanzapine can be a useful long-term strategy for enhancing SRI efficacy, particularly in patients with comorbid bipolar disorder [42].

More recently, Dar et al. (2021) demonstrated that olanzapine (up to 10 mg/day) significantly reduced Y-BOCS scores over six weeks in 60 treatment-resistant patients, comparable to aripiprazole [32].

Altogether, olanzapine appears to be an effective short- to medium-term augmentation strategy in SSRI- or clomipramine-resistant OCD, though its metabolic side-effect profile and limited long-term efficacy warrant careful monitoring.

#### 2.2.4. Quetiapine

Quetiapine, an atypical antipsychotic with dual antagonism at 5-HT2 and D2 receptors, has been investigated as an augmentation strategy in treatment-resistant obsessive-compulsive disorder [6].

Carey et al. (2005) conducted a double-blind, placebo-controlled study in 42 patients unresponsive to 12 weeks of serotonin reuptake inhibitor (SRI) therapy. Participants were randomized to adjunctive quetiapine (up to 300 mg/day) or placebo for 6 weeks. Both groups exhibited significant reductions in Yale-Brown Obsessive-Compulsive Scale scores, with 40% of quetiapine-treated patients and 47.6% of placebo-treated patients meeting responder criteria; however, quetiapine did not demonstrate superiority over placebo, raising uncertainty about its true therapeutic benefit in OCD [43]. Another study by Bogan et al. (2005) investigated adjunctive quetiapine in 30 adults with SRI-resistant OCD during an 8-week, open-label trial. The authors reported a modest mean Y-BOCS reduction at one site (from 27.7 ± 7.0 to 23.3 ± 8.4; responder rate = 31%), but a substantially lower effect at the second site (responder rate = 14%), suggesting only limited benefit under less favorable conditions [44].

Longer-term data from Matsunaga et al. (2009) in a 12-month, open-label trial of 44 SSRI-refractory patients receiving adjunctive quetiapine, risperidone, or olanzapine alongside cognitive-behavioral therapy (CBT) demonstrated significant reductions in Y-BOCS scores (mean ± SD: 29.3 ± 9.9 to 19.3 ± 6.8), though improvements were less pronounced compared to patients responsive to SSRIs plus CBT alone. Patients receiving quetiapine had higher baseline symptom severity and continued to show more severe symptoms at one year. Safety concerns, including weight gain and metabolic effects, were also highlighted [25].

Overall, these findings suggest that quetiapine may be beneficial for some patients with refractory OCD, particularly certain symptom subtypes, but its efficacy remains uncertain and long-term use requires careful monitoring.

### 2.3. Extended-Release Methylphenidate (MPH-ER)

Methylphenidate (MPH), a dopaminergic and noradrenergic reuptake inhibitor, has emerged as a novel augmentation candidate in treatment-resistant OCD [20].

Mudgal et al. (2025) reported 6 patients with OCD unresponsive to SSRIs and prior augmentation strategies. Adjunctive MPH led to significant reductions in Y-BOCS scores (indicating improvement in overall OCD symptom severity) and improvements in cognitive flexibility and impulse control (specific executive functions assessed in the study) [45]. Similarly, Dogan-Sander and Strauß (2021) described a 33-year-old patient with comorbid ADHD and OCD who showed insufficient response to paroxetine and quetiapine; treatment with extended-release MPH 30 mg/day produced marked improvements in both ADHD and OCD symptoms, with symptom worsening upon unsupervised discontinuation and renewed improvement after reinitiation [18].

Zheng et al. (2018) conducted a randomized, double-blind, placebo-controlled trial in 44 patients with treatment-resistant OCD, comparing fluvoxamine plus MPH (36 mg/day) versus fluvoxamine plus placebo. MPH was administered using a fixed titration schedule (18 mg/day increased to 36 mg/day). MPH augmentation yielded significantly greater reductions in Y-BOCS scores (*p* < 0.001), suggesting enhanced therapeutic response. However, because MPH was studied only in combination with fluvoxamine, the generalizability of these findings to other SSRIs or clomipramine remains uncertain [20].

Although preliminary findings suggest MPH-ER may reduce Y-BOCS scores and improve executive control, the overall certainty of evidence remains low due to small sample sizes, heterogeneous populations, and limited long-term follow-up. Moreover, stimulant-related risks (e.g., anxiety exacerbation, insomnia, cardiovascular effects) necessitate careful patient selection and monitoring. At present, MPH-ER should be considered experimental and best positioned as a later-line augmentation option within specialized settings pending larger confirmatory trials.

## 3. Discussion

Across randomized controlled trials, open-label studies, and meta-analytic syntheses, SGAs remain the most consistently supported augmentation strategy for SSRI-resistant OCD. Among these, risperidone and aripiprazole demonstrate the strongest and most reproducible efficacy, whereas olanzapine and quetiapine show more heterogeneous and less robust outcomes. However, response criteria varied across studies, with some trials defining response as ≥25% Y-BOCS reduction and others using ≥35%, which complicates direct comparisons between studies. The evidence base for antipsychotic and MPH-ER augmentation in treatment-resistant OCD includes randomized controlled trials (RCTs), open-label studies, and case reports. RCTs generally provide the highest level of evidence, with rigorous blinding and control conditions, though most sample sizes are modest and follow-up periods short. Open-label studies and case reports offer preliminary or hypothesis-generating insights but are susceptible to bias, including placebo effects, selective reporting, and heterogeneity in dosing or outcome measures. Where possible, we indicate study design, sample size, and risk of bias to contextualize findings and interpret results with caution.

Aripiprazole, a dopamine D2/D3 partial agonist and 5-HT1A partial agonist, represents a mechanistically distinct augmentation option. By stabilizing dopaminergic signaling rather than producing full antagonism, aripiprazole may normalize both hyper- and hypo-dopaminergic states across cortical and subcortical regions [24]. This pharmacodynamic profile may explain its favorable tolerability and its potential advantages in improving executive function and cognitive control, as suggested by several controlled trials.

Risperidone has repeatedly shown superiority to placebo at low doses (typically 1–3 mg/day), with clinically meaningful reductions in Y-BOCS scores in approximately 40–50% of treatment-resistant patients. Its efficacy is thought to derive from potent dopamine D2 receptor antagonism combined with serotonin 5-HT2A receptor blockade, leading to modulation of striatal dopaminergic hyperactivity within cortico-striato-thalamo-cortical (CSTC) circuits [6,7]. Neuroimaging studies suggest that this dopaminergic modulation may reduce pathological habit formation and improve inhibitory control over intrusive thoughts [46,47].

In contrast, olanzapine and quetiapine exhibit inconsistent efficacy across studies. While both agents possess serotonergic and dopaminergic antagonism, their broader receptor profiles and sedative or metabolic adverse effects may limit clinical applicability. Olanzapine has demonstrated short-term benefits in selected patients but carries a substantial risk of weight gain and metabolic dysregulation, which is particularly relevant given the chronic nature of OCD treatment. Quetiapine, despite theoretical appeal, has not consistently outperformed placebo in controlled trials, suggesting that its role should be restricted to carefully selected cases.

### 3.1. Molecular, Genetic, and Pharmacogenomic Perspectives

A major limitation of the existing literature is the relative lack of molecular and pharmacogenomic stratification in augmentation trials. Interindividual variability in response and tolerability to antipsychotic augmentation may be partly explained by genetic variation in drug metabolism and dopamine signaling pathways. For example, risperidone and aripiprazole are primarily metabolized by CYP2D6 and CYP3A4, and polymorphisms in these enzymes can significantly influence plasma concentrations, prolactin elevation, and neurological adverse effects [8,9]. Similarly, variation in dopamine receptor genes (e.g., DRD2, DRD3) and serotonin receptor genes (e.g., HTR2A) may modulate treatment response, although this remains insufficiently explored in OCD populations [6,13]. In addition, carboxylesterase-1 (CES1) variants may alter MPH pharmacokinetics, and emerging evidence from recent genome-wide association studies and polygenic-risk analyses suggests that DRD2 and DRD3 polymorphisms can influence both treatment response and susceptibility to adverse effects, highlighting the potential for pharmacogenomic-guided personalization of augmentation strategies [48,49].

Integrating pharmacogenomic and molecular biomarkers into future trials could enable more personalized augmentation strategies and reduce trial-and-error prescribing. Research recommendations: future RCTs of antipsychotic augmentation should stratify by CYP2D6 metabolizer status; MPH-ER trials should incorporate CES1 genotyping to optimize dosing and reduce variability; larger, multi-center studies should integrate pharmacogenomic and cognitive measures to enable personalized augmentation strategies. At present, routine genotyping is not standard practice in treatment-resistant OCD, as evidence remains preliminary and most clinical laboratories do not yet offer actionable pharmacogenomic panels for antipsychotic or MPH augmentation. Nevertheless, genotyping may be considered in research contexts or for patients with prior unusual drug responses, and future guidelines could incorporate CYP2D6, CYP3A4, CES1, and DRD/HTR polymorphisms once more robust evidence is available.

### 3.2. Extended-Release Methylphenidate: Promise and Limitations

The exploration of MPH-ER as an augmentation strategy represents a novel and conceptually interesting extension of dopaminergic modulation in OCD. Unlike antipsychotics, which primarily reduce striatal dopaminergic activity, MPH increases extracellular dopamine and norepinephrine in prefrontal regions, potentially enhancing executive functions such as attention, response inhibition, and cognitive flexibility [19,50]. These mechanisms may be particularly relevant for OCD patients with prominent executive dysfunction or comorbid attention-deficit/hyperactivity disorder (ADHD).

However, the current evidence base for MPH-ER remains limited. Data are derived mainly from a single small randomized controlled trial, supplemented by case reports and small case series. Sample sizes are modest, follow-up periods are short, and patient populations are heterogeneous. From a molecular perspective, MPH metabolism is strongly influenced by CES1 genetic variants, which may contribute to variability in exposure and clinical response [10].

Given these limitations, MPH-ER should not be viewed as a standard augmentation strategy but rather as an experimental or later-line option within specialized settings. Larger, well-designed trials incorporating cognitive, neurobiological, and pharmacogenomic measures are essential before MPH-ER can be confidently integrated into clinical guidelines.

### 3.3. Practical Sequencing of Augmentation Strategies

**Figure 2:**
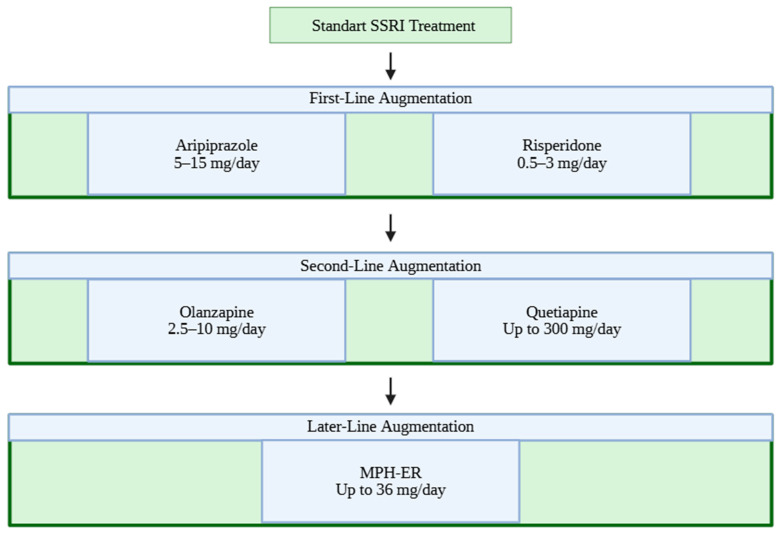
Clinical Practice Algorithm for SSRI-resistant OCD.

In clinical settings, aripiprazole (5–15 mg/day) is preferred in patients at risk of metabolic syndrome or sedation sensitivity, with potential cognitive benefits; risperidone (0.5–3 mg/day) suits those needing stronger D2 blockade without major prolactin concerns. Olanzapine (2.5–10 mg/day) may be considered for comorbid bipolar disorder, insomnia, or SGA failures, but metabolic monitoring is essential, while quetiapine (up to 300 mg/day) is limited to cases needing sedation, given inconsistent efficacy (Figure 2). Extended-release MPH (18–36 mg/day) may be reserved for confirmed ADHD with executive deficits after two SGA failures, with careful monitoring for OCD worsening, insomnia, or cardiovascular effects [51,52].

## 4. Materials and Methods

This narrative review aimed to summarize evidence on pharmacological augmentation strategies for patients with and obsessive-compulsive disorder (OCD). Literature searches were conducted using the PubMed, EBSCO, and Embase databases. The search strategy included the following keywords and their combinations: augmentation strategies, pharmacotherapy, treatment-resistant, antipsychotics, dopamine receptor binding affinity, extended-release methylphenidate (MPH-ER), and obsessive-compulsive disorder (OCD). This allowed the inclusion of studies addressing both augmentation strategies and patients with OCD. Zotero reference management software was used to organize retrieved articles and facilitate selection.

### 4.1. Inclusion Criteria

- Articles published within the last 30 years;
- Studies analyzing augmentation or pharmacological strategies in OCD, including both empirical research and theoretical reviews;
- Studies providing sufficient information to indicate that the participants were adults diagnosed with OCD;
- Publications in English.

### 4.2. Exclusion Criteria

- Articles published more than 30 years ago;
- Articles not directly related to augmentation strategies, pharmacotherapy, or their impact on treatment outcomes in OCD;
- Studies including only minor participants (<18 years old).

### 4.3. Study Selection

After the initial search, duplicates were removed, and titles and abstracts of approximately 100 records were screened for relevance. Although a formal flow diagram was not created, approximately 20 studies were included (Table 1), while 80 studies were excluded due to lack of relevance or insufficient data. Most included studies were small, single-center RCTs, open-label trials, or case series. Many lacked blinding or used variable Y-BOCS response criteria (≥25% vs. ≥35%). These limitations are important for interpreting the robustness and generalizability of findings. We selected a 30-year publication window to capture early haloperidol and SGA augmentation data. Very early SGA studies from the mid-1990s were systematically screened, although most did not meet inclusion criteria. No pediatric/child-adolescent studies were included; therefore, all conclusions pertain to adult populations only.

## 5. Conclusions

1. Antipsychotic augmentation remains a cornerstone strategy for SSRI-resistant OCD, with the strongest evidence supporting risperidone and aripiprazole as first-line augmenting agents.

2. Among first-generation antipsychotics (FGAs), haloperidol shows some efficacy, particularly in patients with comorbid tic disorders or schizotypal traits; however, its clinical use is limited by a narrow therapeutic window and a high risk of extrapyramidal side effects, and it should be reserved for exceptional cases when second-generation antipsychotics (SGAs) are contraindicated.

3. Olanzapine and quetiapine can be considered in specific clinical scenarios; however, evidence is less consistent and adverse-effect burden often restricts clinical applicability.

4. Extended-release methylphenidate (MPH-ER) shows promising preliminary results, particularly in patients with comorbid ADHD and executive function deficits, after failure of at least two SGAs; it should be considered experimental and used only within specialized settings.

5. Clinical caution: all augmentation strategies are off-label; informed consent and close monitoring are mandatory, particularly for metabolic, cardiovascular, and psychiatric adverse effects.

6. Future studies should emphasize standardized response definitions, longer follow-up, and mechanistic stratification of patients (e.g., symptom dimensions, comorbidity profiles, cognitive markers).

## Figures and Tables

**Table 1:** Selected studies.

Study	Sample Size	Intervention and Comparator	Main Outcomes (Y-BOCS)	Response (%)	Treatment Duration
Randomized Controlled Trials (RCTs)					
Zheng et al., 2018 (double-blind, placebo controlled)	N = 44; treatment-refractory OCD	Fluvoxamine 250 mg/day + MPH-ER (18 → 36 mg/day) vs. fluvoxamine 250 mg/day + placebo	Greater Y-BOCS reduction in MPH-ER group from week 2 onward (F = 8.16, *p* = 0.005)	Partial response (≥25% Y-BOCS): 36.4% vs. 4.6%; full response (≥35%): 22.7% vs. 0%	8 weeks
McDougle et al., 2000 (double-blind, placebo-controlled)	N = 36; SRI-resistant OCD	Adjunctive risperidone (mean dose 2.2 mg/day) vs. placebo	Y-BOCS reduced by 31.8% (27.4 ± 5.4 → 18.7 ± 8.3) with risperidone; no significant change with placebo (*p* < 0.001)	50% (risperidone completers) vs. 0% (placebo); responders showed 51.6% Y-BOCS reduction	6 weeks
Bystritsky et al., 2004 (double-blind, placebo-controlled)	N = 26; SRI-refractory OCD	Olanzapine augmentation (up to 20 mg/day) + ongoing SRI vs. placebo	Y-BOCS change: olanzapine −4.2 (SD 7.9); placebo +0.54 (SD 1.31)	6/13 (46%) olanzapine vs. 0/13 placebo (≥25% Y-BOCS)	6 weeks
Sayyah et al., 2012 (double-blind, placebo-controlled)	N = 39; treatment-resistant OCD	Aripiprazole 10 mg/day + ongoing SSRI vs. placebo + ongoing SSRI	Y-BOCS decreased from 22.21 ± 4.6 → 15.42 ± 5.1 in Aripiprazole group; 24.12 ± 6.1 → 23.12 ± 5.1 in placebo	8/15 (53%) ari-piprazole vs. 3/17 (17.6%) placebo (≥25% Y-BOCS reduction)	12 weeks
Muscatello et al., 2011 (double-blind, placebo-controlled)	N = 30; treatment-resistant OCD	Aripiprazole 15 mg/day + stable SRI/clomipramine vs. placebo + SRI/clomipramine	Y-BOCS decreased significantly in aripiprazole group (obsessions, *p* = 0.007; compulsions, *p* = 0.001; total, *p* < 0.0001)	11/16 (68.7%) partial response (≥25% Y-BOCS reduction); 4/16 (25%) full re-sponse (≥35% reduction)	16 weeks
Maina et al., 2008 (single-blind)	N = 50; treatment-resistant OCD (from 96 screened)	Risperidone (1–3 mg/day; mean 2.1 mg/day) + ongoing SRI vs. olanzapine (2.5–10 mg/day; mean 5.3 mg/day) + ongoing SRI	Both groups showed significant reductions in Y-BOCS total (paired *t*-test: t = 7.588 for risperidone, t = 7.456 for olanzapine, *p* < 0.001)	Risperidone: 11/25 (44%), Olanzapine: 12/25 (48%) (≥35% Y-BOCS)	8 weeks (after 16-week open SRI trial)
Carey et al., 2005 (double-blind, placebo-controlled)	N = 42; SRI-resistant OCD	Quetiapine augmentation + ongoing SRI (flexible dose; mean dose at week 6: 168.8 ± 120.8 mg/day) vs. placebo augmentation + ongoing SRI	Significant Y-BOCS im-provement within both groups (quetiapine *p* < 0.0001; placebo *p* = 0.001); no significant difference between groups at endpoint (*p* = 0.636)	≥25% Y-BOCS reduction: quetiapine 40% (8/20) vs. placebo 47.6% (10/21)	6 weeks
Hollander et al., 2003 (double-blind, placebo-controlled)	N = 16; SRI-resistant OCD	Risperidone augmentation + ongoing SRI (0.5–3.0 mg/day; mean 2.25 ± 0.86 mg/day) vs. placebo augmentation + ongoing SRI	Mean Y-BOCS decreased from 29.20 ± 5.73 → 23.10 ± 8.33 (19.0%) on risperidone vs. 29.33 ± 2.80 → 28.00 ± 7.31 (4.6%) on placebo; between-group difference not statistically significant (*p* = 0.198)	≥25% Y-BOCS reduction: risperidone 40% (4/10) vs. placebo 0% (0/6); *p* = 0.115	8 weeks
Assarian et al., 2016 (double-blind, placebo-controlled)	N = 100; SSRI-refractory OCD	Aripiprazole augmentation + SSRI (mean dose 5 mg/day) vs. risperidone (1.5 mg/day) augmentation + SSRI (mean dose 1.5 mg/day) therapy	Y-BOCS baseline: aripiprazole 25.02 ± 4.46, risperidone 25.26 ± 4.17; follow-up: aripiprazole 16.24 ± 4.41, risperidone 20.00 ± 4.45; aripiprazole showed greater reduction (*p* < 0.001)	N/A	12 weeks
Open-label/Long-term/Pilot studies					
Dar et al., 2021 (open-label)	N = 115; SRI-resistant subgroup N = 60	Olanzapine (2.5–10 mg/d), aripiprazole (5–15 mg/d), or L-methylfolate (15 mg/d) added to SRI	Olanzapine and Aripiprazole over 6 weeks (*p* < 0.001); no significant change with L-methylfolate (Y-BOCS *p* = 0.150)	Responder rates ≥ 35% Y-BOCS reduction reported; olanzapine and aripiprazole superior to L-methylfolate	6-week open-label augmentation after 12-week SRI run-in
Bogetto et al., 2000 (open-label)	N = 23; fluvoxamine-refractory OCD	Olanzapine 5 mg/day added to ongoing fluvoxamine (300 mg/day)	Y-BOCS: baseline 26.8 ± 3.0 → follow-up 18.9 ± 5.9; significant decrease (F = 43.811, *p* = 0.0005)	10/23 (43.5%) ≥35% Y-BOCS reduction	12 weeks
Bogan et al., 2005 (open-label)	N = 30; treatment-resistant OCD (S1 = 16; S2 = 14)	Quetiapine augmentation (25 → 200 mg/day) added to ongoing SRI; mean dose: S1 = 169 ± 57 mg/day; S2 = 116 ± 72 mg/day)	Site 1: Y-BOCS 27.7 ± 7.0 → 23.3 ± 8.4 (*p* = 0.01); Site 2: 27.1 ± 4.3 → 25.5 ± 4.7 (not significant change)	≥25% Y-BOCS reduction: Site 1 = 31% (5/16); Site 2 = 14% (2/14)	8 weeks
Matsunaga et al., 2009 (long-term)	N = 44 (SSRI-refractory OCD) vs. N = 46 (SSRI responders)	Atypical antipsychotic (olanzapine 5.1 ± 3.2 mg/day, risperidone 3.1 ± 1.9 mg/day, quetiapine 60 ± 37.3 mg/day) + SSRI + CBT vs. SSRI + CBT	SSRI-refractory: Y-BOCS 29.3 ± 9.9 → 19.3 ± 6.8; SSRI responders: 25.8 ± 11.4 → 13.7 ± 4.6	>50% improvement occurred in both groups (more than 35% Y-BOCS reduction); no group difference	1 year
Marazziti et al., 2005 (long-term)	N = 26; SSRI-resistant OCD	Olanzapine augmentation (2.5–10 mg/day) + ongoing SRI vs. prior SRI monotherapy	Y-BOCS at 1 year: 29.3 ± 6.1 → 18.0 ± 3.3	17/26 (~65%) (at least 35% in the total baseline Y-BOCS)	1 year
Delle Chiaie et al., 2011 (pilot)	N = 20; OCD patients resistant to SSRIs or clomipramine	Aripiprazole augmentation (5–20 mg/day) vs. ongoing SSRI/clomipramine monotherapy	Significant improvement in Y-BOCS (t = 13.146, df = 19, *p* = 0.0001)	Full response (≥35% Y-BOCS reduction): 16/20 (80%); partial response (≥25% Y-BOCS): 2/20 (10%); non-responders: 2/20 (10%)	12 weeks
Ak et al., 2011 (pilot)	N = 30; SRI-resistant OCD	Aripiprazole augmentation added to ongoing SRIs (flexible dose, mean 15.9 ± 7.9 mg/day)	Significant improvement in Y-BOCS (32.0 ± 6.3 → 24.0 ± 8.1; Z = 4.2, *p* < 0.05)	7/23 completed patients (30.4%) met ≥30% Y-BOCS improvement	10 weeks
Case reports/Case series					
Study	Sample size	Intervention and comparator		Main outcomes	
Izci et al., 2016 (case series)	N = 5; treatment-resistant OCD	Aripiprazole augmentation (10–30 mg/day) added to clomipramine vs. previous monotherapy		Good clinical outcomes observed across cases	
Dogan-Sander and Strauß, 2021 (case report)	Single case (33-year-old patient with comorbid OCD + ADHD)	MPH-ER added to sertraline + quetiapine vs. SRI monotherapy		Improvement in both ADHD and OCD symptoms	
Weiss et al., 1999 (case series)	N = 10; SSRI-refractory OCD	Olanzapine (1.25 mg/day–20 mg/day) augmentation added to ongoing SSRI		Responders to olanzapine augmentation showed rapid and sustained improvement in OCD symptoms	
Mudgal et al., 2025 (case series)	N = 6; treatment-resistant OCD	Methylphenidate (20–50 mg/day) added to ongoing SRI treatment		Significant reduction in Y-BOCS in all cases	

Abbreviations: (1) OCD—Obsessive-Compulsive Disorder; (2) ADHD—Attention-Deficit/Hyperactivity Disorder; (3) Y-BOCS—Yale-Brown Obsessive-Compulsive Scale; (4) SRI—Serotonin Reuptake Inhibitor; (5) SSRI—Selective Serotonin Reuptake Inhibitor; (6) MPH-ER—Extended-Release Methylphenidate; (7) CBT—Cognitive-Behavioral Therapy; (8) RCT—Randomized Controlled Trial.

## Data Availability

No new data were created or analyzed in this study. Data sharing is not applicable.
